# Genetic structure of an endangered species *Ormosia henryi* in southern China, and implications for conservation

**DOI:** 10.1186/s12870-023-04231-w

**Published:** 2023-04-26

**Authors:** Chengchuan Zhou, Shiqi Xia, Qiang Wen, Ying Song, Quanquan Jia, Tian Wang, Liting Liu, Tianlin Ouyang

**Affiliations:** 1 Identification and Evaluation Center for Forest Germplasm Resources in Jiangxi Province, Jiangxi Academy of Forestry, Nanchang, China; 2 Jiangxi Provincial Forestry Science and Technology Experiment Center, Ganzhou, China

**Keywords:** *Ormosia henryi*, Population structure, Genetic diversity, Genotyping by sequencing, Conservation

## Abstract

**Background:**

The evergreen broadleaved forest (EBLF) is an iconic vegetation type of East Asia, and it contributes fundamentally to biodiversity-based ecosystem functioning and services. However, the native habitat of EBLFs keeps on decreasing due to anthropogenic activities. *Ormosia henryi* is a valuable rare woody species in EBLFs that is particularly sensitive to habitat loss. In this study, ten natural populations of *O. henryi* in southern China were sampled, and then genotyping by sequencing (GBS) was applied to elucidate the standing genetic variation and population structure of this endangered species.

**Results:**

In ten *O. henryi* populations, 64,158 high-quality SNPs were generated by GBS. Based on these markers, a relatively low level of genetic diversity was found with the expected heterozygosity (*He*) ranging from 0.2371 to 0.2901. Pairwise *F*_*ST*_ between populations varied from 0.0213 to 0.1652, indicating a moderate level of genetic differentiation. However, contemporary gene flow between populations were rare. Assignment test and principal component analysis (PCA) both supported that *O. henryi* populations in southern China could be divided into four genetic groups, and prominent genetic admixture was found in those populations located in southern Jiangxi Province. Mantel tests and multiple matrix regression with randomization (MMRR) analyses suggested that isolation by distance (IBD) could be the possible reason for describing the current population genetic structure. In addition, the effective population size (*Ne*) of *O. henryi* was extremely small, and showed a continuous declining trend since the Last Glacial Period.

**Conclusions:**

Our results indicate that the endangered status of *O. henryi* is seriously underestimated. Artificial conservation measures should be applied as soon as possible to prevent *O. henryi* from the fate of extinction. Further studies are needed to elucidate the mechanism that leading to the continuous loss of genetic diversity in *O. henryi* and help to develop a better conservation strategy.

**Supplementary Information:**

The online version contains supplementary material available at 10.1186/s12870-023-04231-w.

## Background

The evergreen broadleaved forest (EBLF) is the typical vegetation biome in the subtropical regions of China with a range from 22 to 34°N latitude and 99 to 123°E longitude. Considering that the other areas in the same latitude were covered by desert or semi-desert, this biome type is unique owing to its dependence on East Asian monsoon [1]. As this region was not covered by large ice sheets during the Last Glacial Maximum (LGM), many rare or relict plant species have been preserved, making it a hotspot for biodiversity conservation and research [2, 3]. However, natural EBLFs have greatly diminished due to anthropogenic deforestation and the unpredictable extreme climates in recent years seem to increase the potential risk of biodiversity loss. The degradation of habitats caused by these factors has led to the endangerment of many rare species in EBLFs [4]. Although the evergreen broadleaved forests are predicted to expand in the next 100 years according to the representative concentration pathways (RCPs) scenarios under the framework of the Coupled Model Intercomparison Project Phase 5 (CMIP5) [5], the fate of these rare and endangered plant species in EBLFs is still under debate. As an important component of EBLFs, rare and endangered plants are more vulnerable to population loss due to their small population size and low genetic diversity [6]. Therefore, there is an urgent need for a robust assessment of the standing genetic variation and evolutionary potential of the endangered EBLF components to help develop better conservation strategies, especially for those species with high utility value.

Single nucleotide polymorphisms (SNPs) are the most abundant and universal variations across the genome, thus making them very useful markers for population genetic analysis [7, 8]. With the rapid development in next generation sequencing technologies, a variety of reduced-representation genome sequencing (RRGS) methods have been developed, making it possible for non-model species to generate thousands of genome-wide SNP markers without a reference genome [9]. Among these RRGS methods, genotyping by sequencing (GBS) is a flexible, rapid and cost-effective genomic approach combining both marker discovery and genotyping and has been widely used to investigate genetic diversity and population structure of non-model species [10–12]. GBS better detect subtle changes in population structure than traditional markers do, which allows a more comprehensive description of the patterns of genetic variation in non-model species [10–12].

The genus *Ormosia* Jacks. (Fabaceae, Papilionoideae) comprises about 150 species of trees and exhibits a striking disjunct geographical distribution between the New World- and Asian and Australasian wet tropics and subtropics [13]. It is a moderately large genus of particular importance in tropical or subtropical broadleaved forests, with most species generally growing as evergreen (or rarely deciduous) trees [14, 15]. China is the northernmost distribution of the genus *Ormosia*, and there are approximately 35 *Ormosia* species distributed in southern China. Among these species, *O. henryi* is a rare and endangered evergreen tree species with multiple usages. It is highly prized not only for the durable and attractive wood, but also as an excellent ornamental plant [16, 17]. *O. henryi* was recently found to be a new bioactive resource with potential antidepressant activity [18]. However, during the past several decades, the natural populations of *O. henryi* in China have dramatically declined due to over-logging, habitat fragmentation and difficulties in regeneration. *O. henryi* has been classified as a national second-class protected wild plant in China and was evaluated as Vulnerable (VU) by IUCN. Previous studies of *O. henryi* focused on its bioactive ingredients, medical usages and tissue culture [18–20]. By far, we still know little about its genetic variation, population structure or demographical history, which would lead to poor efficiency in conservation, restoration and utilization. Given the lack of a reference genome and molecular markers for *O. henryi*, genotyping-by-sequencing (GBS) method was adopted in this study. We identified genome-wide SNPs from ten natural populations of *O. henryi* in southern China to address the following questions: (1) to assess the genetic diversity and population structure of *O. henryi*, (2) to evaluate the impact of geography and environment on genetic differentiation of *O. henryi* and (3) to infer the demographic history of *O. henryi*. All these results will provide a molecular basis for the conservation of *O. henryi* natural populations and provide a technical reference for their rational utilization.

## Results

### Reference survey genome and SNP discovery

After removing reads with adapters and reads with low quality, a total of 190,771,231 pairs of reads were obtained, reaching 57.23 Gb of clean data. A total of 4,066,523 contigs were assembled by SOAPdenovo software, and the maximum and N50 contig lengths were 54,197 bp and 656 bp, respectively (Additional file 1: Table S2). The GC content of contigs was 35.27%. The Further assembly generated 3,866,097 scaffolds, and the maximum and N50 scaffold lengths were 59,305 bp and 762 bp, respectively. The assembled survey genome of *O. henryi* was 1,364,689,951 bp in length (including N) and served as the reference genome for GBS data analysis.

A total of 1764 million reads, 264.67 Gb clean data were generated by GBS. The mapping rates of all samples ranged from 34.20% to 41.55%, and the effective sequencing depth ranged from 4.19 to 7.97 × (Additional file 1: Table S3). With our parameter settings, 331,206 SNPs were identified from 129 samples by bcftools. After filtering for minor allele frequency (MAF ≥ 0.05), missing rate (≤ 0.2), and read depth (DP ≥ 4), 64,158 high-quality SNPs were obtained for subsequent data analysis. In the outlier detection analysis, only six SNPs were identified as putative selected sites by BayeScan with a *q*-value threshold of 0.05, while no outlier SNPs were detected with a *q*-value threshold of 0.01 (Additional file 2: Fig. S1). Therefore, most SNPs were putatively neutral and used for population genetic analyses after filtering.

### Genetic diversity, population differentiation and contemporary gene flow

At the species level, the observed heterozygosity (*Ho*), expected heterozygosity (*He*), and nucleotide diversity (π) were 0.1460, 0.1594 and 0.1132, respectively, suggesting a low level of genetic diversity in *O. henryi* populations. At the population level, genetic diversity based on the three statistics showed different trends of ranking. The *Ho* values ranged from 0.2282 (XF) to 0.2874 (SX) with an average of 0.2675, while the *He* values ranged from 0.2371 (XF) to 0.2901 (AY) with an average of 0.2669. The nucleotide diversity (π) varied between 0.1216 (YK) and 0.1429 (JH) with an average of 0.1312. The results showed that PIC values varied from 0.2023 to 0.2335 with an average of 0.2213. The inbreeding coefficient (*F*_*IS*_) values among populations were close to zero, ranging from -0.0232 to 0.0224 (Table 1).

**Table 1:** Summary of genetic diversity for 10 *O. henryi* populations

Population	*n*	*Ho*	*He*	π	*F*_*IS*_	PIC	*Ne* (95% CI)
Jianghua (JH)	13	0.2639	0.2729	0.1429	0.0224	0.2268	58.1 (40.5–96.8)
Xinfeng (XF)	15	0.2282	0.2371	0.1321	0.0185	0.2028	43.5 (18.4-inf)
Anyuan (AY)	8	0.2831	0.2901	0.1401	0.0143	0.2335	45.7 (13.7-inf)
Ninghua (NH)	15	0.2646	0.2564	0.1284	-0.0181	0.2140	6.9 (2.3–30.9)
Matoushan (MTS)	15	0.2565	0.2601	0.1283	0.0079	0.2167	7.0 (1.4–41.6)
Shunchang (SC)	13	0.2736	0.2653	0.1311	-0.0203	0.2183	6.0 (1.9–35.3)
Shaxian SX	10	0.2874	0.2825	0.1293	-0.0054	0.2321	1.8 (0.7–19.8)
Jianou (JO)	10	0.2695	0.2743	0.1356	0.0094	0.2259	14.3 (2.1-inf)
Longquan (LQ)	15	0.2626	0.2552	0.1230	-0.0146	0.2150	3.4 (1.5–26.7)
Yongkang (YK)	15	0.2855	0.2751	0.1216	-0.0232	0.2278	3.0 (1.6–12.9)
All	129	0.1460	0.1594	0.1316	0.0554	0.1587	3.4 (2.6–13.6)

*Ho* observed heterozygosity, *He* expected heterozygosity, π nucleotide diversity, *F*_*IS*_ inbreeding coefficients, *PIC* Polymorphism information content, *Ne* effective population size, inf refers to *Ne* ‘infinite’

Pairwise *F*_*ST*_ values between *O. henryi* populations varied from 0.0213 to 0.1652, with an average value of 0.0918, suggesting a moderate level of genetic differentiation among these *O. henryi* populations (Table 2). The geographically most distant JH and YK populations had the highest *F*_*ST*_ value, while the geographically closest AY and XF populations had the lowest *F*_*ST*_ value.

**Table 2:** Pairwise *F*_*ST*_ values between different populations of *O. henryi* collected in southern China

	JH	XF	AY	NH	MTS	SC	SX	JO	LQ	YK
JH	-									
XF	0.0914	-								
AY	0.0990	0.0213	-							
NH	0.1358	0.0827	0.0860	-						
MTS	0.1316	0.0701	0.0741	0.0769	-					
SC	0.1337	0.0757	0.0748	0.0780	0.0630	-				
SX	0.1430	0.0851	0.0881	0.0822	0.0714	0.0687	-			
JO	0.1115	0.0577	0.0611	0.0639	0.0477	0.0495	0.0531	-		
LQ	0.1420	0.0791	0.0878	0.0954	0.0674	0.0869	0.0918	0.0591	-	
YK	0.1652	0.1140	0.1192	0.1397	0.1248	0.1318	0.1423	0.1124	0.0951	-

Contemporary gene flow analysis detected significant migration rates from XF to AY, from MTS to NH, and from SC to SX (Table 3). All other migration rates were very low (< 0.05) and not significant from zero, suggesting that overall contemporary gene flow between *O. henryi* populations was limited.

**Table 3:**
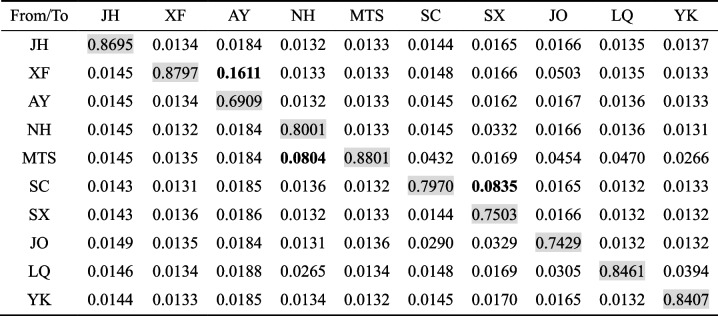
Recent migration rates between populations estimated using BA3-SNPs

The source populations are listed in the first column. Values in bold indicate significant estimates of migration rates where the calculated 95% credible sets do not include zero. Shaded values along the diagonal indicate the mean proportion of non-migrants within a population

### Population genetic structure

**Fig. 1:**
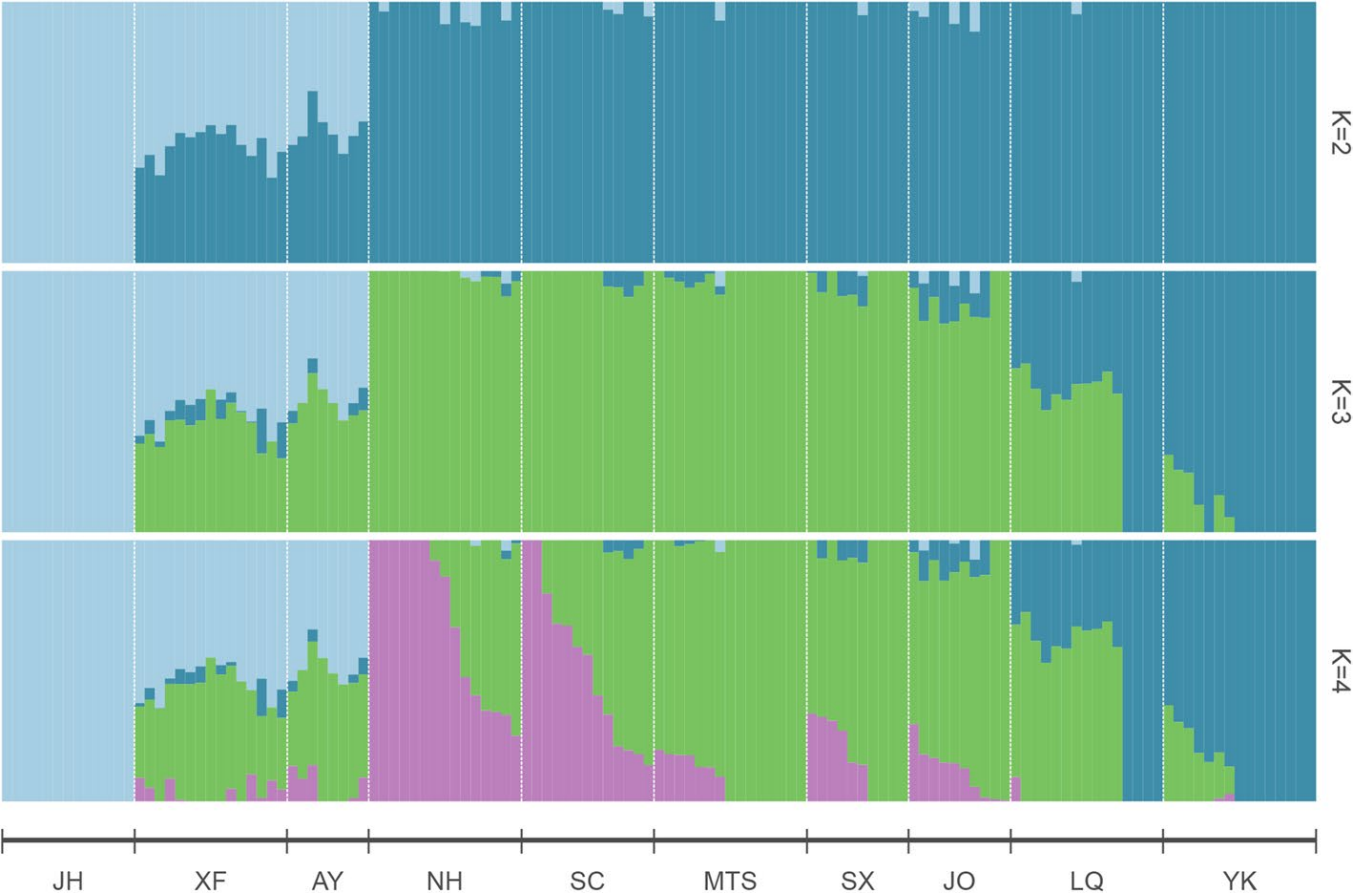
ADMIXTURE results of the 129 *O. henryi* individuals based on the dataset of putative neutral SNPs with K = 2, K = 3 and K = 4

Based on the putative neutral SNP dataset, ADMIXTURE identified K = 4 as the most appropriate number according to the CV error (Additional file 2: Fig. S2). Under K = 2, the 129 *O. henryi* individuals from 10 populations were clustered into three groups. The first group was comprised of all the individuals from JH, the second group was comprised of all individuals from XF and AY, and the third group consisted of the remaining individuals. Under K = 3 and K = 4, all 129 individuals were divided into four groups, the first and second group were maintained unchanged, but the third group was split into one large group with all the individuals from NH, SC, MTS, SX and JO, and one small group with all the individuals from LQ and YK. Regardless of the K values, clear genetic admixtures were observed in AY and XF populations from southern Jiangxi province (Fig. 1).

**Fig. 2:**
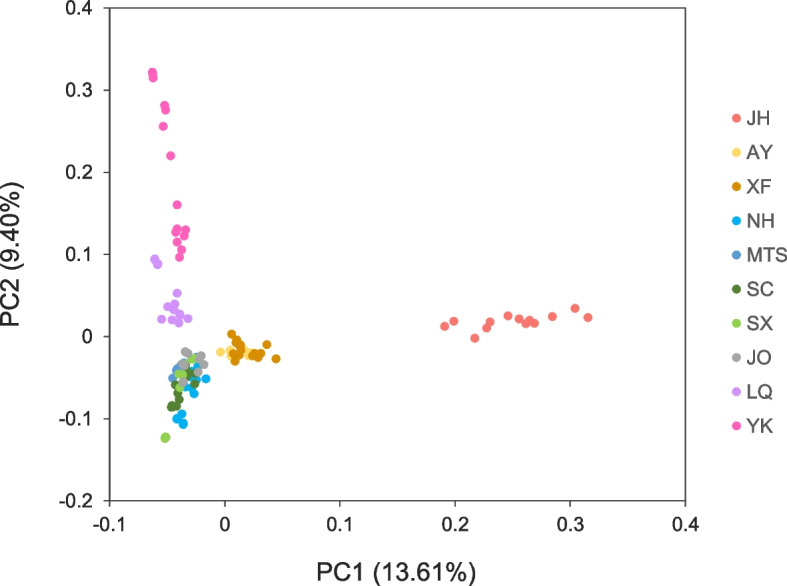
Clustering of *O. henryi* populations based on principal component analysis (PCA). Each point represents an individual colored according to the sampling site

**Fig. 3:**
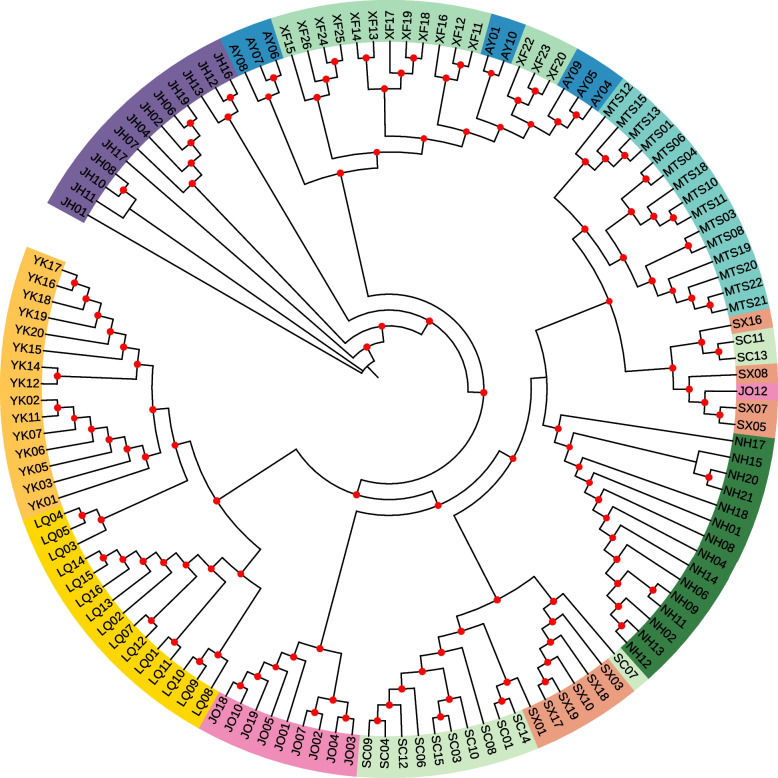
The neighbor-joining (NJ) tree of all the 129 *O. henryi* individuals based on the dataset of putative neutral SNPs. The red dot indicates the branch support value of bootstraps > 50%

To better understand the population structure of *O. henryi*, PCA and phylogenetic tree construction were performed. PCA results supported the division of four genetic groups by ADMIXTURE analysis under K = 3 or 4. Two principal components PC1 and PC2 accounted for 13.61% and 9.40% of total variability, respectively. At PC1, individuals from JH were clearly separated from the other individuals, and individuals from AY and XF were slightly separated from the remaining individuals. At PC2, individuals from NH, SC, MTS, SX and JO were mixed but distinct from individuals from LQ and YK (Fig. 2). The unrooted NJ tree of 129 *O. henryi* individuals also supported the division of four genetic groups. There were clear boundaries among the four genetic groups, as individuals from the same genetic group clustered together on the NJ tree (Fig. 3). AMOVA analysis revealed that 89.92% of the total variation was found within populations, and only 4.22% and 5.86% were found among groups and among populations within groups, respectively (Table 4).

**Table 4:** Analysis of molecular variance (AMOVA) among groups, among populations within groups and within populations in *O. henryi*

Source of variation	*df*	Sum of squares	Variation components	Percentage of variation(%)	Fixation indices	*p* value
Among groups	3	42,634.32	122.746	4.22	*F*_*CT*_: 0.0422	*p* < 0.0001
Among populations within groups	6	41,441.56	170.578	5.86	*F*_*SC*_: 0.0612	*p* < 0.0001
Within populations	248	649,205.41	2617.764	89.92	*F*_*ST*_: 0.1008	*p* < 0.0001
Total	257	733,281.29	2911.088			

### Effects of IBD and IBE on genetic differentiation

**Fig. 4:**
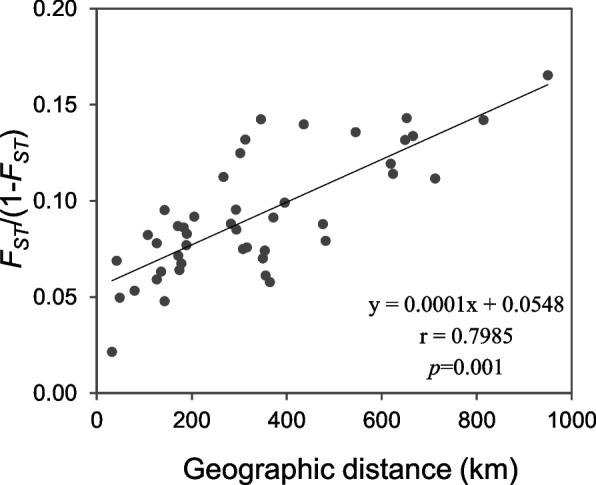
The significant correlation between genetic distance and geographic distance among 10 *O. henryi* populations found by Mantel test

Mantel tests showed a significant correlation between pairwise *F*_*ST*_/(1-*F*_*ST*_) and geographic distance for the 10 *O. henryi* populations (*r* = 0.7985, *p* = 0.001; Fig. 4), but no significant correlation was detected either between pairwise *F*_*ST*_/(1-*F*_*ST*_) and environmental distance (*r* = 0.2742, *p* = 0.157) or between geographic distance and environmental distance (*r* = 0.1511, *p* = 0.271). The results of partial Mantel tests were similar. Pairwise *F*_*ST*_/(1-*F*_*ST*_) displayed a significant correlation with geographic distance after controlling for environmental distance (*r* = 0.7095, *p* = 0.001), but no significant correlation was observed between pairwise *F*_*ST*_/(1-*F*_*ST*_) and environmental distance after controlling for geographical distance (*r* = 0.2177,* p* = 0.177). The multiple matrix regression with randomization (MMRR) analysis revealed the relative contributions of IBD and IBE, and further confirmed strong and significant effects of IBD (*β*_D_ = 0.5698, *p* = 0.0001) but weak and non-significant effects of IBE (*β*_E_ = 0.0587, *p* = 0.3120) on population genetic differentiation.

### Demographic history of *O. henryi*

**Fig. 5:**
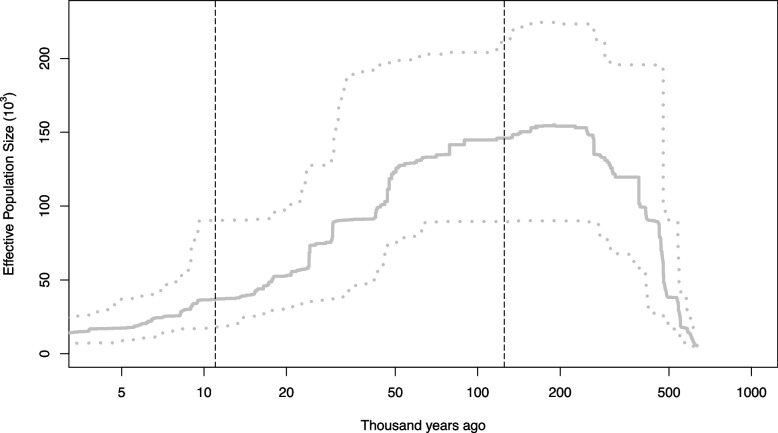
Inference of demographic history of the entire *O. henryi* population using Stairway plot 2. The grey solid line represents the median effective population size (*Ne*) and the grey dash lines represent the 2.5 and 97.5 percentiles of *Ne*, respectively. The area between the two black dashed lines represents the Last Glacial Period

We estimated a contemporary effective population size (*Ne*) of 3.4 (95% CI: 2.6—13.6) for all the *O. henryi* individuals. The *Ne* estimates of JH, XF and AY populations were larger than 40, while the *Ne* estimates of other populations were below 20 (Table 1). Demographic analysis by Stairway Plot 2 showed that *O. henryi* experienced a population expansion of about 0.2–0.6 Mya and began to decline during the Last Glacial Period. About 50, 000 years ago, the effective population size of *O. henryi* declined sharply, and no recovery trend of *Ne* was observed (Fig. 5, Additional file 2: Fig. S3).

## Discussion

### Genetic diversity and differentiation

Generally, species or populations with higher genetic diversity are more capable of adapting to environmental changes [21, 22]. In this study, a relatively low level of genetic diversity was found in *O. henryi* at both species and population levels. The observed heterozygosity (*Ho*) and expected heterozygosity (*He*) of *O. henryi* were 0.1460 and 0.1594 at the species level respectively, which were lower than those of some other tree species revealed by SNP markers, for example, *Cinnamomum camphora* (*Ho* = 0.319, *He* = 0.322, [23]), *Quercus acutissima* (*Ho* = 0.2319, *He* = 0.2272, [24]) and *Eucalyptus regnans* (*Ho* = 0.22, *He* = 0.232, [25]). Notably, the genetic diversity of *O. henryi* (nSSR, *Ho* = 0.340, *He* = 0.492, unpublished data) is much lower than that of *O. hosiei* (nSSR, *Ho*: 0.560–0.712, *He*: 0.723–0.835, [26]), which is also classified as a national second-class protected wild plant in China. As one of the most northerly distributed and cold-tolerant species in *Ormosia*, *O. hosiei* occurs in our sampling area as well. *O. hosiei* was evaluated as Endangered (EN) by IUCN, which is supposed to be rarer than *O. henryi* (Vulnerable, VU). The endangered status of *O. hosiei* with high genetic diversity is likely due to the recent population declines caused by human activities, and the high level of genetic diversity observed in *O. hosiei* could be a transitory state [17, 26, 27]. Our demographic analysis indicated that the effective population size (*Ne*) of *O. henry* began to decline during the Last Glacial Period (LGP) and the contemporary *Ne* estimate was very low at the species level. Thus, the relatively low genetic diversity of *O. henryi* appears to be a result of both evolutionary history and human-induced population declines.

Our results showed that the rankings of genetic diversity of different populations based on *Ho* and *He* were similar but quite different from that based on nucleotide diversity (π). Considering the three statistics (*Ho*, *He* and π) together, the AY population had relatively high genetic diversity despite only eight individuals being found in our field investigation. Contemporary *Ne* is usually smaller than the real population size [28]. A relatively large contemporary *Ne* was estimated in AY population, suggesting that there may be more undiscovered individuals of *O. henryi* in AY population.

Many forest tree species show a high level of genetic variation within populations, with relatively low genetic differentiation among populations [29]. As expected, AMOVA results showed that the genetic variation of *O. henryi* was mainly maintained within populations, with a moderate and significant genetic differentiation detected among populations (*F*_*ST*_ = 0.1008, *p* < 0.001). It is widely accepted that IBD and IBE are two main models of population genetic differentiation, but IBD and IBE are not mutually exclusive and can jointly contribute to population divergence [30, 31]. In this study, IBD was strongly supported by the Mantel tests and MMRR analysis, suggesting that gene flow is geographically limited in *O. henryi*. Seeds of *O. henryi* are dispersed over short distances mainly by gravity [32]. However, due to the low germination rate under natural conditions, *O. henryi* does not regenerate well in the understory and seedlings are rarely seen [32]. Seeds of *O. henryi* may also attract arboreal frugivorous birds and rodents for long-distance seed dispersal like the other tree species of *Ormosia* [33, 34], but effective gene flow between *O. henryi* populations has not developed, resulting in isolation. Populations with low genetic diversity are very sensitive to genetic drift. The factors above may enhance the effect of IBD. In addition, physical barriers such as mountains, rivers or man-made landscapes can facilitate genetic divergence by obstructing dispersal among populations [35, 36]. In southern China, there are several major mountains (e.g., Wuyi Mountains, Nanling Mountains) that might serve as physical barriers to prevent the gene exchange between populations. Strong IBD patterns have been found in many other tree species in this region, such as *Castanopsis fargesii* [37], *Schima superba* [38] and *Sassafras tzumu* [39]*.* On the other hand, no significant effects of IBE were detected by either Mantel tests or MMRR analysis. A strong pattern of IBE is typically interpreted as evidence that divergent selection associated with environmental heterogeneity is maintaining population differentiation [40]. In consist with the insignificant effects of IBE, we found only a few SNPs possibly under selection in the outlier detection analysis, suggesting that the genetic differentiation of *O. henryi* populations was mainly driven by neutral processes like genetic drift, but not local adaptation to different climate conditions.

### Population structure and gene flow

Understanding the population structure of a threatened species can help identify conservation units and develop effective conservation strategies [41]. Significant population structure of *O. henryi* was identified by the ADMIXTURE analysis, PCA and phylogenetic analysis, supporting the division of four groups. Echoing the IBD pattern, the population structure of *O. henryi* also exhibited variation with geographic distance. The JH population located in the range of Nanling Mountains is geographically isolated from other populations. The genetic composition of JH population was distinct from other populations regardless of the K value. The NH, SC, MTS, SX and JO populations from the third group located in the adjacent areas of Wuyi Mountains were closely related, and some individuals even clustered with individuals from other populations on the phylogenetic tree (e.g., SC11, SC13, JO12 were clustered with some individuals from SX population). According to the results of PCA and ADMIXTURE analysis, the LQ population from the fourth group was genetically more similar to the populations in the third group than the YK population. The AY and XF populations in southern Jiangxi province were observed with clear genetic admixtures, suggesting that southern Jiangxi province may be a transition zone between eastern and western populations.

Gene flow plays an important role in shaping the spatial genetic structure [42]. Small, isolated populations often suffer from genetic drift and reduced gene flow, which is more likely to generate a prominent population structure [43]. Contemporary gene flow analysis showed that significant recent migrate rates (m) were detected between only a few nearby populations (from XF to AY, from MTS to NH, and from SC to SX). Therefore, the genetic admixtures observed in the AY and XF populations were more likely due to historical gene flow rather than contemporary gene flow. Given the poor dispersal ability of *O. henryi*, it probably relies primarily on short-distance, gradual dispersal. Without habitat destruction, there can be high levels of gene flow among *O. henryi* populations. Once the population connectivity is broken by the processes such as habitat fragmentation and human activities, gene flow will be restricted and a significant population structure will generate.

### Implications for conservation of *O. henryi*

The exploration of genetic diversity and population structure can provide vital information for the conservation and management of threatened species [44]. Our population genetic analysis suggests the possible worsening of the *O. henryi* endangerment situation. The contemporary *Ne* estimates of *O. henryi* were much lower than the numbers generally suggested in the 50/500 rule. A *Ne* of 50 is recommended to avoid short-term inbreeding depression, and a *Ne* of 500 to maintain the long-term evolutionary potential of a species [45, 46]. The demographic analysis also revealed the sustained declining trend of *Ne* since the LGP. Accordingly, only a small number of individuals were found in most natural populations during our field investigation. Many isolated trees were found distributed in fragmented habitats such as roadsides, riversides and beside villages. The low *Ne* estimates, along with the low level of genetic diversity and the observed small population size are warning signs that *O. henryi* is at increased risk of genetic erosion and should therefore be subject to conservation intervention urgently. Moreover, a moderate level of genetic differentiation and strong IBD pattern were detected in the ten *O. henryi* populations from southern China. If the conservation and management of *O. henryi* is not strengthened in time, genetic differentiation among *O. henryi* populations will further increase, eventually leading to a more endangered status.

*O. henryi* has attracted much attention for its valuable timber, high ornamental value, and potential medicinal value. In recent years, *O. henryi* has been gradually used in the transformation of low-quality and inefficient forests such as pure fir forests and pure pine forests in southern China. However, there are still some problems in the conservation and utilization of *O. henryi* such as inadequate *ex-situ* conservation, arbitrary selection of provenances in the reforestation process and lack of good varieties. Considering the underestimated endangered status of *O. henryi*, urgent actions should be taken for both *in-situ* and *ex-situ* conservation. In addition, genetic assessment of natural populations outside the highly suitable areas should be conducted to determine a more comprehensive conservation strategy. Based on the findings of this study, several conservation measures are suggested here: (i) In situ conservation should be further strengthened. Priority should be given to the protection of large populations with high genetic diversity in natural forest stands. Maintaining their existing natural habitats is one of the most important ways to protect them. Our results have divided the ten populations in highly suitable areas into four genetic groups, which can be protected as separate evolutionary units. (ii) *Ex-situ* conservation should be carried out for the long-term survival of *O. henryi*. The establishment of germplasm resource nurseries and low-temperature seed banks is recommended. Individuals from different genetic groups especially superior trees should be collected to ensure genetic representativeness. (iii) Reintroduction and restoration programs are necessary for increasing population size and preventing further isolation. Seed germination of *O. henryi* is limited by its hard, dense seed coat and inhibitory substances [32, 47], while seedlings are slow-growing and poorly resistant to drought and frost [48]. Therefore, artificial propagation is recommended. The seedlings can be transplanted to the land near the original populations as they grow up, to help the population recover. Furthermore, the selection of provenances should follow the principle of 'right tree for right place', taking into account factors such as genotypes, forest stands, pests, soil texture and plant growth-promoting rhizobacteria in order to improve the survival rate of seedlings.

## Conclusions

In this study, we performed a genome-wide population genetic investigation on natural populations of *O. henryi* in southern China. Based on the GBS method, we found a low level of genetic diversity, a moderate level of genetic differentiation and significant genetic structures in natural* O. henryi* populations. Isolation by distance (IBD) is considered as the main reason for the current population genetic structure. Continuous declining trend of the effective population size was suggested, which occurred during the Last Glacial Period. The results from the current genomic data combined with our field investigation suggest that the endangered status of *O. henryi* is seriously underestimated. Both ex situ and in situ conservation measures should be applied as soon as possible to prevent *O. henryi* from the fate of extinction. To gain more complete scenario, we call for more studies to decipher the mechanism that leads to the continuous loss of genetic diversity in *O. henryi*. Our population genomic study provides valuable information for the conservation and management of *O. henryi* and contributes to the further understanding of preserving the biodiversity in EBLFs. As our sampling area did not cover the entire range of *O. henryi*, the genetic diversity and differentiation of *O. henryi* may have been underestimated in this study. In addition, we lacked an in-depth understanding of the important biological characteristics of *O. henryi*, such as mating system, seed dispersal and natural population regeneration. Therefore, further studies are needed to clarify the endangerment mechanism of *O. henryi*.

## Methods

### Sample collection and DNA extraction

**Fig. 6:**
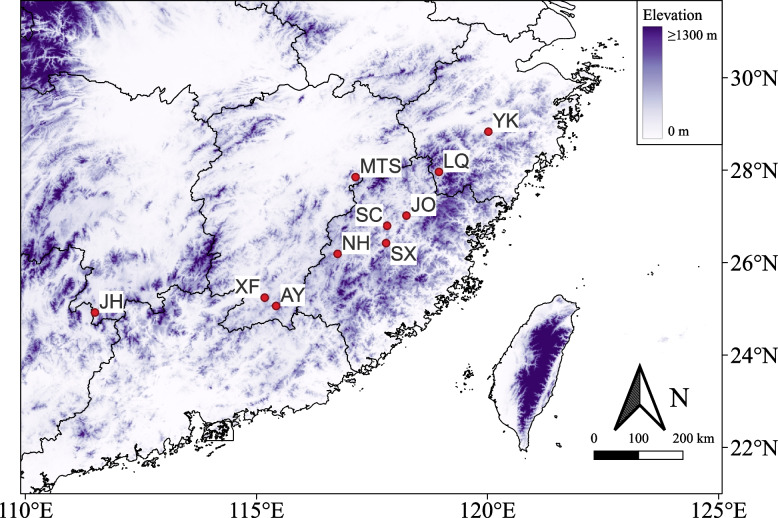
Sampling locations of 10 *O. henryi* populations investigated in southern China

Results from our preliminary ecological niche modeling of *O. henryi* suggest that, under current climatic conditions, the core distribution of *O. henryi* is mainly concentrated in Jiangxi, Hunan, Fujian, Zhejiang provinces in southern China [49]. To understand the genetic structure of *O. henryi* in its core distribution range, 129 *O. henryi* individuals were collected from 10 populations in the four provinces of southern China (Fig. 6, Additional file 1: Table S1). In each population, only mature plants were sampled with a minimum distance of 50 m. The samples were carefully identified by Dr. Faxin Yu from Institute of Biological Resources, Jiangxi Academy of Sciences based on the descriptions in Flora of China, a voucher specimen was deposited in the Herbarium of Jiangxi Academy of Forestry with an accession number ZGSZOHE0006. Fresh young leaves from each healthy *O. henryi* individual were collected and quickly frozen with liquid nitrogen. Samples were taken back to the lab and frozen at -80℃ until DNA extraction.

Total genomic DNA was extracted by the modified CTAB method [50]. DNA purity and concentration were checked by the NanoDrop 1000 spectrophotometer (NanoDrop Technologies, Wilmington, DE, USA). DNA interity was then evaluated on 1% (w/v) agarose gel by electrophoresis.

### Reference genome construction and genotyping by sequencing (GBS)

One *O. henryi* sample from XF population was selected for genome survey sequencing. Qualified genomic DNA was randomly sheared into fragments using an ultrasonicator (Covaris, Woburn, MA, USA). A paired-end DNA library was prepared and sequenced with Illumina HiSeq X-ten platform. After removing reads with adapters and reads with low quality, the survey genome assembly was performed using SOAPdenovo [51] software with the K-mer set at 41.

The GBS libraries were prepared following the method developed by Elshire et al. [52]. Two restriction enzymes (*MseI* and *NlaIII*) were used to digest previously extracted DNA. Barcoded adapters were ligated to the digested fragments, and multiplex amplification by PCR was performed. All the samples were mixed, and the desired fragments were selected for GBS library construction in Novogene Co., Ltd (Beijing, China). Paired-end sequencing was performed on an Illumina HiSeq sequencing platform.

### Processing of sequencing data and SNP calling

The barcoded adapter sequences of raw reads were removed by the process_radtags program in the STACKS v2.1 software [53]. Raw reads were further filtered and trimmed with the FASTX toolkit v0.0.01 (http://hannonlab.cshl.edu/fastx_toolkit/). The obtained clean reads were aligned to the previously assembled survey genome of *O. henryi* using bwa v0.7.8 [54] with the following settings: mem -t 4 -k 32 -M. The resulting sam files were converted to bam files by samtools v 1.7 [55]. Picard v1.119 (https://broadinstitute.github.io/picard/) was used to sort bam files by coordinates and mark PCR duplicates. SNP calling was performed using bcftools v1.7 [56] with default parameters. The raw SNP dataset was further filtered with a minor allele frequency of at least 0.05 (–maf 0.05), a genotyping rate of at least 80% (–max-missing 0.8) and a minimum depth of 4 (–minDP 4) by vcftools v0.1.13 [57].

### Population genetic analysis

To fulfil neutrality assumptions in population genetic analyses, we detected potential SNPs under selection using the Bayesian method as implemented in BayeScan v2.1 [58]. To assesss genetic diversity of *O. henryi*, observed heterozygosity (*Ho*) and expected heterozygosity (*He*) for each population was estimated by Arlequin v3.5 [59], and nucleotide diversity (π) for each population was calculated by vcftools v0.1.13. The inbreeding coefficient (*F*_*IS*_) and polymorphism information content (PIC) was calculated according to the formula [60, 61]. Pairwise genetic differentiation (*F*_*ST*_) between populations were calculated by Arlequin v3.5. The contemporary gene flow among populations was estimated by BA3-SNPs program [62] with 10 million iterations, a burn-in of 1 × 10^6^, and a sampling frequency of 1000. Mixing parameters for allele frequencies, inbreeding coefficients and migration rates were adjusted to achieve acceptance rates between 0.2 and 0.6 as recommended by the software's manual. Five independent runs with different random seeds were performed. Migration rates were considered significant if the 95% credible sets did not include zero.

The population genetic structure was investigated using phylogenetic tree construction, principal component analysis (PCA) and the model-based evolutionary clustering approach as implemented in ADMIXTURE v1.2 [63]. An unrooted neighbor-joining (NJ) tree of all the 129 samples based on all the neutral SNPs was inferred using megacc software with 1000 bootstraps [64]. Principal component analysis (PCA) was performed by the –pca function in PLINK v1.9 software [65]. In the ADMIXTURE analysis, the pre-defined genetic clusters (K) were set from 2 to 10, and different random seeds were set for 10 repeated runs. The most appropriate K value was selected according to the lowest value of cross-validation (CV) error rate, and the results were visualized by R package Pophelper v2.3.1 [66].

AMOVA analysis was carried out by Arlequin v3.5 to quantify the genetic variation among and within the genetic groups inferred by the population structure analyses. The significance levels for the variance components were tested using 1,000 permutation steps.

### Isolation by distance (IBD) and isolation by environment (IBE)

To investigate the roles of geographic and environmental factors in shaping the spatial genetic differentiation, we calculated: (a) the correlation between environmental and geographic distance, (b) isolation by distance (IBD) and (c) isolation by environment (IBE). The geographic distance matrix was calculated based on the GPS coordinates of sampling sites using GenAlex v6.51 [67]. A total of 19 bioclimate variables for each sampling site were obtained from the WorldClim database averaged for the years 1970–2000 at 30 s spatial resolution (~ 1 km) (https://worldclim.org/, [68]). To avoid multicollinearity, Pearson's correlation coefficients between bioclimate variables were calculated by "cor" function in R. Highly correlated variables (|r|> 0.8) were removed and 10 bioclimate variables were kept for IBE analysis (Additional file 1: Table S4). The environmental distance matrix was calculated by first scaling and centering the 10 selected bioclimate variables to account for differences in magnitude, then calculating pairwise Euclidean distances between sampling sites using R package vegan [69]. Pairwise genetic distance *F*_*ST*_ /(1- *F*_*ST*_) between populations was calculated by Arlequin v3.5. Mantel tests were performed to assess the associations between genetic distance, geographic distance and environmental distance with 9999 permutations in R package vegan. The relative role of geography (IBD) and environment (IBE) on population divergence was also evaluated using multiple matrix regression with randomization (MMRR) implemented in the "MMRR" function of R [70]. MMRR allows quantification of how a dependent variable (genetic distance) responds to changes in explanatory variables (geographic and environmental distance). Regression coefficients of IBD (*β*_D_) and IBE (*β*_E_) were determined after 9999 permutations.

### Demographic history inference

Contemporary effective population size (*Ne*) for each population was estimated using the linkage disequilibrium method in NeEstimator v.2.1 [71] with the random mating model and the minor allele frequency cutoff of 1/(2n) (n = sample size). The demographic history of *O. henryi* was inferred using Stairway Plot 2 [72] based on site frequency spectrum (SFS). The folded SFS was constructed by a custom python script easySFS.py with a down projection method (https://github.com/isaacovercast/easySFS). The mutation rate for *O. henryi* was taken from literature-based estimates for genome-wide mutation rates and was eventually set at 4e-9 per site per year [73–75]. The generation time was set as 7 years, which was estimated from our long-term field observation of the time from seeds to flowering in Jiangxi Academy of Forestry. Other parameters were set following the manual's instructions, and bootstrap iterations of 200 were implemented.

## Supplementary Information

**Additional file 1: Table S1.** Sampling information of *Ormosia henryi* populations. **Table S2.** Statistics of the *O. henryi* genome assembly. **Table S3.** Summary of GBS data. **Table S4.** Ten bioclimate variables used in this study.

**Additional file 2: Fig S1.** Potential SNPs under selection identified by BayeScan. The vertical solid line represents the threshold for being under selection after correction with false discovery rate (0.05). Six SNPs colored in red were probably under selection. **Fig S2.** Cross-validation (CV) errors of 10 repeat runs under different K values. **Fig. S3**. Demographic history of three genetic groups of* Ormosia henryi* inferred by Stairway Plot 2. The x-axis indicates the time before the present, and the y-axis represents the historical effective population size. The first genetic group were not used in the demographic history analysis due to the small number of individuals.

## Data Availability

The plant materials were collected from natural populations in the geographic distribution of *Ormosia henryi*. The raw reads files can be accessed on NCBI Sequence Read Archive (SRA) under the BioProject number of PRJNA909598.

## Abbreviations

GBS: Genotyping by sequencing
PCA: Principal component analysis
MMRR: Multiple matrix regression with randomization
RRGS: Reduced representation genome sequencing
*Ho*: Observed heterozygosity
*He*: Expected heterozygosity
π: Nucleotide diversity
PIC: Polymorphism information content
*F*_*IS*_: Inbreeding coefficient
SNP: Single nucleotide polymorphism
AMOVA: Analysis of molecular variance
IBD: Isolation by distance
IBE: Isolation by environment
CV: Cross-validation
