# Supplementary material for: Genetic structure of an endangered species Ormosia henryi in southern China, and implications for conservation
Source: BMC Plant Biol. 2023 Apr 26;23:220. doi: 10.1186/s12870-023-04231-w (PMC10131447; doi:10.1186/s12870-023-04231-w)
Supplement: Supplementary file 2 — Additional file 2: Fig S1. Potential SNPs under selection identified by BayeScan. The vertical solid line represents the threshold for being under selection after correction with false discovery rate (0.05). Six SNPs colored in red were probably under selection. Fig S2. Cross-validation (CV) errors of 10 repeat runs under different K values. Fig. S3. Demographic history of three genetic groups of Ormosia henryi inferred by Stairway Plot 2. The x-axis indicates the time before the present, and the y-axis represents the historical effective population size. The first genetic group were not used in the demographic history analysis due to the small number of individuals. [file 12870_2023_4231_MOESM2_ESM.docx]

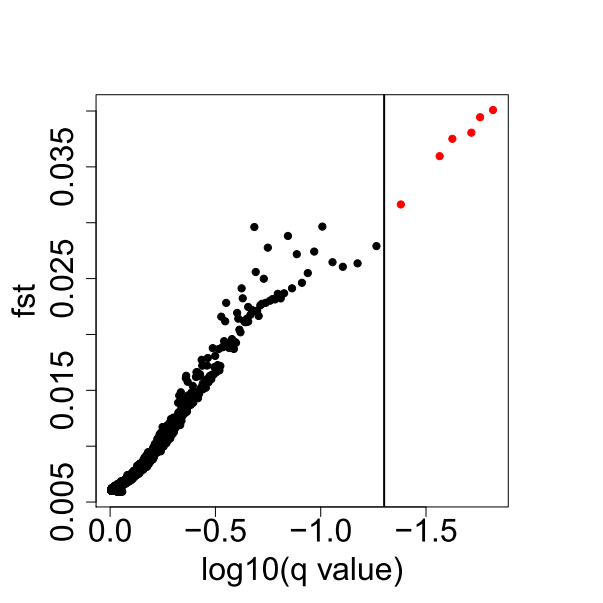


**Fig S1**. Potential SNPs under selection identified by BayeScan. The vertical solid line represents the threshold for being under selection after correction with false discovery rate (0.05). Six SNPs colored in red were probably under selection.


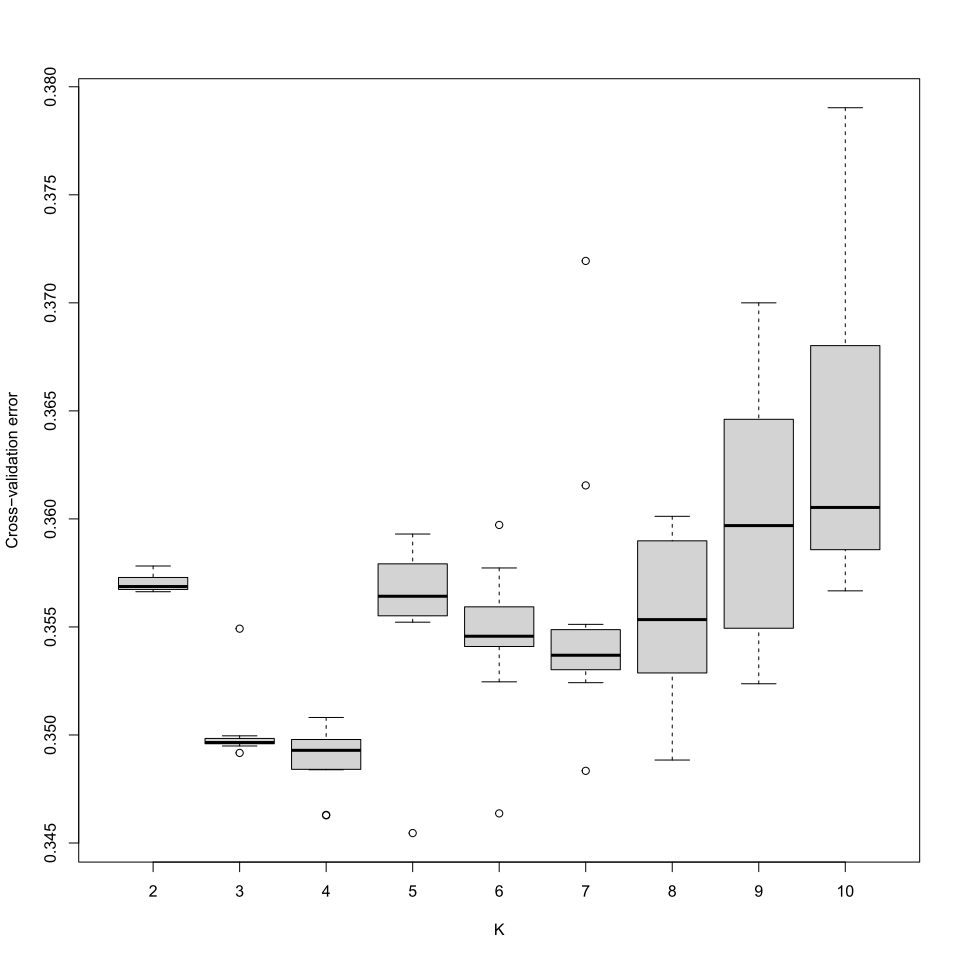


**Fig S2**. Cross-validation (CV) errors of 10 repeat runs under different K values.


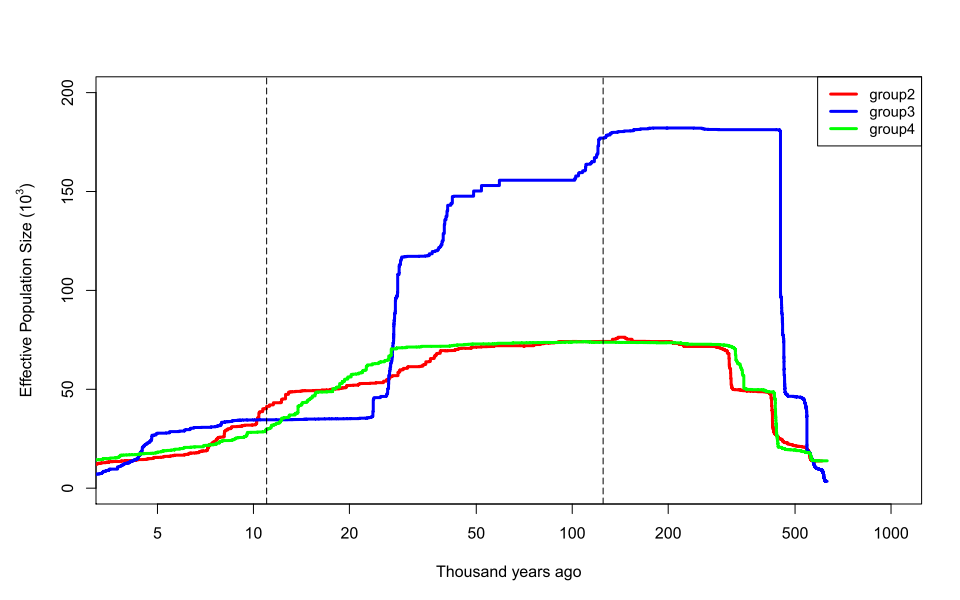


**Fig. S3**. Demographic history of three genetic groups of *Ormosia henryi* inferred by Stairway Plot 2. The x-axis indicates the time before the present, and the y-axis represents the historical effective population size. The first genetic group were not used in the demographic history analysis due to the small number of individuals.
